# Circulating cytokines, chemokines and adhesion molecules in normal pregnancy and preeclampsia determined by multiplex suspension array

**DOI:** 10.1186/1471-2172-11-59

**Published:** 2010-12-02

**Authors:** András Szarka, János Rigó, Levente Lázár, Gabriella Bekő, Attila Molvarec

**Affiliations:** 1 First Department of Obstetrics and Gynecology, Semmelweis University, Budapest, Hungary; 2 Central Laboratory, Semmelweis University, Budapest, Hungary

## Abstract

**Background:**

Preeclampsia is a severe complication of pregnancy characterized by an excessive maternal systemic inflammatory response with activation of both the innate and adaptive arms of the immune system. Cytokines, chemokines and adhesion molecules are central to innate and adaptive immune processes. The purpose of this study was to determine circulating levels of cytokines, chemokines and adhesion molecules in normal pregnancy and preeclampsia in a comprehensive manner, and to investigate their relationship to the clinical features and laboratory parameters of the study participants, including markers of overall inflammation (C-reactive protein), endothelial activation (von Willebrand factor antigen) and endothelial injury (fibronectin), oxidative stress (malondialdehyde) and trophoblast debris (cell-free fetal DNA).

**Results:**

Serum levels of interleukin (IL)-1beta, IL-1 receptor antagonist (IL-1ra), IL-2, IL-4, IL-6, IL-8, IL-10, IL-12p40, IL-12p70, IL-18, interferon (IFN)-gamma, tumor necrosis factor (TNF)-alpha, transforming growth factor (TGF)-beta1, interferon-gamma-inducible protein (IP)-10, monocyte chemotactic protein (MCP)-1, intercellular adhesion molecule (ICAM)-1 and vascular cell adhesion molecule (VCAM)-1 were measured in 60 preeclamptic patients, 60 healthy pregnant women and 59 healthy non-pregnant women by multiplex suspension array and ELISA. In normal pregnancy, the relative abundance of circulating IL-18 over IL-12p70 and the relative deficiency of the bioactive IL-12p70 in relation to IL-12p40 might favour Th2-type immunity. Although decreased IL-1ra, TNF-alpha and MCP-1 concentrations of healthy pregnant relative to non-pregnant women reflect anti-inflammatory changes in circulating cytokine profile, their decreased serum IL-10 and increased IP-10 levels might drive pro-inflammatory responses. In addition to a shift towards Th1-type immunity (expressed by the increased IL-2/IL-4 and IFN-gamma/IL-4 ratios), circulating levels of the pro-inflammatory cytokines IL-6 and TNF-alpha, the chemokines IL-8, IP-10 and MCP-1, as well as the adhesion molecules ICAM-1 and VCAM-1, were raised in preeclampsia compared with healthy pregnancy, resulting in an overall pro-inflammatory systemic environment. Increased IP-10, MCP-1, ICAM-1 and VCAM-1 concentrations of preeclamptic patients showed significant correlations with blood pressure values, renal and liver function parameters, as well as with CRP, malondialdehyde, von Willebrand factor antigen and fibronectin levels.

**Conclusions:**

According to our findings, preeclampsia was associated with an overall pro-inflammatory systemic environment. Elevated amounts of pro-inflammatory cytokines, chemokines and adhesion molecules in the maternal circulation might play a central role in the excessive systemic inflammatory response, as well as in the generalized endothelial dysfunction characteristics of the maternal syndrome of preeclampsia.

## Background

Preeclampsia, characterized by hypertension and proteinuria developing after midgestation, is a severe complication of human pregnancy with a worldwide incidence of 2-10%. It is one of the leading causes of maternal, as well as perinatal morbidity and mortality, even in developed countries. Despite intensive research efforts, the etiology and pathogenesis of preeclampsia are not completely understood. Increasing evidence suggests that an excessive maternal systemic inflammatory response to pregnancy with activation of both the innate and adaptive arms of the immune system is involved in the pathogenesis of the disease [1,2]. The development of preeclampsia is influenced by both genetic and environmental risk factors, suggesting its multifactorial inheritance [3-8].

An important feature of systemic inflammation in preeclampsia is the absence of Th2 skewness characteristic for healthy pregnancy, and thus the predominance of Th1-type immunity. Saito et al. reported firstly that the percentage of Th1 cells and the ratios of Th1/Th2 were significantly higher, while the percentage of Th2 cells was significantly lower in the peripheral blood in preeclampsia than in the third trimester of normal pregnancy [9]. In another study, this group observed increased production of interleukin (IL)-2, interferon (IFN)-γ and tumor necrosis factor (TNF)-α by peripheral blood mononuclear cells (PBMCs) in preeclampsia and, interestingly, positive correlations between mean blood pressure and concentrations of Th1 cytokines [10]. The shift to a predominant Th1-type immunity in preeclampsia was reinforced by other experiments on intracellular cytokine measurements in peripheral blood T (both helper and cytotoxic) cells and NK cells, as well as by assessment of cytokine secretion levels of PBMCs isolated from preeclamptic patients [11-14]. However, the studies on circulating levels of cytokines in normal pregnancy and preeclampsia yielded conflicting results [15,16]. The discrepancies may be due to different techniques used for cytokine detection, differences in the ethnicity of the study populations, disease severity or sample sizes.

The aim of this study was to determine circulating levels of cytokines, chemokines and adhesion molecules in a comprehensive manner involving a large number of healthy non-pregnant and pregnant women and preeclamptic patients. We also measured several markers of processes involved in the pathogenesis of preeclampsia, and investigated whether serum cytokine, chemokine and adhesion molecule levels were related to the clinical characteristics and laboratory parameters of the study participants, including markers of overall inflammation (C-reactive protein), endothelial activation (von Willebrand factor antigen) and endothelial injury (fibronectin), oxidative stress (malondialdehyde) and trophoblast debris (cell-free fetal DNA).

## Methods

### Study patients

Our study was designed using a case-controlled approach. Sixty preeclamptic patients, 60 healthy pregnant women with uncomplicated pregnancies and 59 healthy non-pregnant women were involved in the study. The study participants were enrolled in the First Department of Obstetrics and Gynecology and in the Department of Obstetrics and Gynecology of Kútvölgyi Clinical Center, at the Semmelweis University, Budapest, Hungary. All women were Caucasian and resided in the same geographic area in Hungary. Exclusion criteria were multifetal gestation, chronic hypertension, diabetes mellitus, autoimmune disease, angiopathy, renal disorder, maternal or fetal infection and fetal congenital anomaly. The women were fasting, none of the pregnant women were in active labor, and none had rupture of membranes. The healthy non-pregnant women were in the early follicular phase of the menstrual cycle (between cycle days 3 and 5), and none of them received hormonal contraception.

Preeclampsia was defined by increased blood pressure (≥140 mmHg systolic or ≥90 mmHg diastolic on ≥2 occasions at least 6 hours apart) that occurred after 20 weeks of gestation in a woman with previously normal blood pressure, accompanied by proteinuria (≥0.3 g/24 h or ≥1 + on dipstick in the absence of urinary tract infection). Blood pressure returned to normal by 12 weeks postpartum in each preeclamptic study patient. Preeclampsia was regarded as severe if any of the following criteria was present: blood pressure ≥160 mmHg systolic or ≥110 mmHg diastolic, or proteinuria ≥5 g/24 h (or ≥3 + on dipstick). Pregnant women with eclampsia or HELLP syndrome (hemolysis, elevated liver enzymes, and low platelet count) were not enrolled in this study. Early onset of preeclampsia was defined as onset of the disease before 34 weeks of gestation (between 20 and 33 completed gestational weeks). Fetal growth restriction was diagnosed if the fetal birth weight was below the 10^th ^percentile for gestational age and gender, based on Hungarian birth weight percentiles [17].

The study protocol was approved by the Regional and Institutional Committee of Science and Research Ethics of the Semmelweis University, and written informed consent was obtained from each patient. The study was conducted in accordance with the Declaration of Helsinki.

### Biological samples

Blood samples were taken from an antecubital vein into plain, as well as EDTA- or sodium citrate anticoagulated tubes, and then centrifuged at room temperature with a relative centrifugal force of 3000 *g *for 10 minutes. The aliquots of serum and plasma were stored at -80°C until the analyses.

### Laboratory methods

Serum levels of IL-1β, IL-1 receptor antagonist (IL-1ra), IL-2, IL-4, IL-6, IL-8, IL-10, IL-12p40, IL-12p70, IL-18, IFN-γ, TNF-α, interferon-γ-inducible protein (IP)-10, monocyte chemotactic protein (MCP)-1, intercellular adhesion molecule (ICAM)-1 and vascular cell adhesion molecule (VCAM)-1 were measured by multiplex suspension array (Bio-Plex, Cat. No. X500317TGY and XF0000ZGAI) on a Bio-Plex 200 analyzer (Bio-Rad Laboratories, Hercules, California, USA). Levels of transforming growth factor (TGF)-β1 in maternal sera were assessed by ELISA (DRG International, Mountainside, New Jersey, USA, Cat. No. EIA-1864). Standard laboratory parameters (clinical chemistry) and C-reactive protein (CRP) levels were determined by an autoanalyzer (Cobas Integra 800, Roche, Mannheim, Germany) using the manufacturer's kits. Plasma von Willebrand factor antigen (VWF:Ag) levels were quantified by ELISA (Dakopatts, Glostrup, Denmark), while plasma fibronectin concentration by nephelometry (Dade Behring, Marburg, Germany), according to the manufacturer's instructions. After extracting DNA with the silica adsorption method, the amount of cell-free fetal DNA in maternal plasma was determined in patients with male newborns by quantitative real-time PCR analysis of the sex-determining region Y (SRY) gene, as we described previously [18]. Plasma malondialdehyde levels were measured by the thiobarbituric acid-based colorimetric assay [19].

### Statistical analysis

The normality of continuous variables was assessed using the Shapiro-Wilk's *W*-test. As the continuous variables were not normally distributed, nonparametric statistical methods were used. To compare continuous variables between two groups, the Mann-Whitney *U*-test was applied, whereas to compare them among multiple groups, the Kruskal-Wallis analysis of variance by ranks test was performed. Multiple comparisons of mean ranks for all groups were carried out as post-hoc tests. The Fisher exact and Pearson χ^2 ^tests were used to compare categorical variables between groups. The Spearman rank order correlation was applied to calculate correlation coefficients.

Statistical analyses were performed using the following software: STATISTICA (version 8.0; StatSoft, Inc., Tulsa, Oklahoma, USA) and Statistical Package for the Social Sciences (version 15.0 for Windows; SPSS, Inc., Chicago, Illinois, USA). For all statistical analyses, p < 0.05 was considered statistically significant.

In the article, data are reported as median (25-75 percentile) for continuous variables and as number (percentage) for categorical variables.

## Results

### Patient characteristics

The clinical characteristics of the study participants are described in Table 1. There was no statistically significant difference in terms of age among the study groups. Furthermore, no significant differences were observed in gestational age at blood collection and the percentage of primiparas between preeclamptic patients and healthy pregnant women. However, all of the other clinical features presented in Table 1 differed significantly among our study groups. Fetal growth restriction was absent in healthy pregnant women, whereas the frequency of this condition was 18.3% in the preeclamptic group. Twenty-one women had severe preeclampsia and 5 patients experienced early onset of the disease.

**Table 1:** Clinical characteristics of healthy non-pregnant and pregnant women and preeclamptic patients

	Healthy non-pregnant women (n = 59)	Healthy pregnant women (n = 60)	Preeclamptic patients (n = 60)
Age (years)	28 (23-35)	30 (28-32)	29 (26-32)

BMI at blood draw (kg/m^2^)	20.8 (19.6-22.9)	25.8 (24.3-27.9)^b^	29.9 (26.9-33.3)^b,d^

Smokers	14 (23.7%)	0 (0%)^b^	3 (5.0%)^a^

Primiparas	n.a.	37 (61.7%)	38 (63.3%)

Systolic blood pressure at blood draw (mmHg)	115 (110-120)	110 (107-120)	162 (155-180)^b,d^

Diastolic blood pressure at blood draw (mmHg)	80 (70-80)	70 (60-80)^b^	100 (97-110)^b,d^

Gestational age at blood draw (weeks)	n.a.	36 (36-37)	37 (36-39)

Gestational age at delivery (weeks)	n.a.	39 (38-40)	38 (37-39)^d^

Fetal birth weight (grams)	n.a.	3450 (3150-3700)	3125 (2450-3475)^d^

Fetal growth restriction	n.a.	0 (0%)	11 (18.3%)^d^

Data are presented as median (25-75 percentile) for continuous variables and as number (percentage) for categorical variables

n.a.: not applicable; BMI: body mass index

^a ^p < 0.05 versus healthy non-pregnant women

^b ^p < 0.001 versus healthy non-pregnant women

^c ^p < 0.05 preeclamptic patients versus healthy pregnant women

^d ^p < 0.001 preeclamptic patients versus healthy pregnant women

### Laboratory parameters

The laboratory parameters of the study subjects are displayed in Table 2. As can be seen in the table, there were significant differences in most of the measured laboratory parameters among the three study groups except for serum aspartate aminotransferase (AST) activity. Circulating levels of cytokines, chemokines and adhesion molecules are shown in Table 3. Apart from serum IL-1β and TGF-β1 levels, all of the measured inflammatory variables differed significantly among our study groups.

**Table 2:** Laboratory parameters of healthy non-pregnant and pregnant women and preeclamptic patients

	Healthy non-pregnant women (n = 59)	Healthy pregnant women (n = 60)	Preeclamptic patients (n = 60)
Serum BUN level (mmol/l)	4.1 (3.5-4.8)	2.8 (2.0-3.3)^b^	3.5 (2.7-4.2)^a,c^

Serum creatinine level (μmol/l)	66 (61-72)	49 (42-56)^b^	63 (55-71)^d^

Serum bilirubin level (μmol/l)	9.3 (6.6-12.4)	5.4 (4.0-6.8)^b^	7.3 (5.7-8.9)^a,c^

Serum AST activity (U/l)	17 (15-20)	19 (17-21)	19 (15-25)

Serum ALT activity (U/l)	14 (12-17)	12 (10-15)^a^	16 (11-23)^c^

Serum LDH activity (U/l)	154 (128-170)	158 (138-169)	192 (153-225)^b,d^

Serum CRP level (mg/l)	0.7 (0.5-1.8)	3.6 (1.7-6.6)^b^	6.8 (2.7-12.1)^b,c^

Plasma VWF:Ag level (%)	70.0 (60.2-87.3)	152.6 (112.7-199.0)^b^	184.8 (139.9-243.1)^b,c^

Plasma fibronectin level (g/l)	n.m.	0.37 (0.31-0.47)	0.58 (0.41-0.82)^d^

Plasma malondialdehyde level (nmol/ml)	n.m.	15.4 (8.8-18.6)	18.3 (15.6-20.4)^c^

Plasma cell-free fetal DNA level (pg/μl)	n.m.	0.002 (0.0-0.172)^†^	0.082 (0.033-0.292)^‡,c^

Data are presented as median (25-75 percentile)

n.m.: not measured; BUN: blood urea nitrogen; AST: aspartate aminotransferase; ALT: alanine aminotransferase; LDH: lactate dehydrogenase; CRP: C-reactive protein; VWF:Ag: von Willebrand factor antigen; DNA: deoxyribonucleic acid

^† ^n = 19

^‡ ^n = 33

^a ^p < 0.05 versus healthy non-pregnant women

^b ^p < 0.001 versus healthy non-pregnant women

^c ^p < 0.05 preeclamptic patients versus healthy pregnant women

^d ^p < 0.001 preeclamptic patients versus healthy pregnant women

**Table 3:** Serum levels (pg/ml) of cytokines, chemokines and adhesion molecules in healthy non-pregnant and pregnant women and preeclamptic patients

	Healthy non-pregnant women (n = 59)	Healthy pregnant women (n = 60)	Preeclamptic patients (n = 60)
IL-1β	24.5 (23.0-28.0)	27.0 (23.0-31.5)	28.0 (23.0-34.0)

IL-1ra	8.0 (6.7-11.0)	6.0 (5.0-7.0)^b^	18.0 (11.0-27.5)^b,d^

IL-2	5.0 (4.2-6.0)	4.0 (4.0-5.0)^a^	7.5 (5.5-12.0)^b,d^

IL-4	3.0 (2.0-3.0)	2.0 (2.0-2.0)^b^	3.0 (3.0-4.0)^b,d^

IL-6	6.0 (5.0-8.0)	7.0 (5.0-9.0)	15.5 (12.0-32.0)^b,d^

IL-8	23.0 (18.2-37.5)	24.5 (16.0-68.5)	78.0 (35.0-273)^b,d^

IL-10	43.7 (32.7-60.5)	15.7 (14.0-19.0)^b^	23.0 (18.0-35.0)^b,d^

IL-12p40	119 (109-140)	136 (118-168)^a^	185 (153-215)^b,d^

IL-12p70	12.0 (9.0-15.0)	5.0 (4.0-5.0)^b^	6.0 (5.0-8.0)^b,d^

IL-18	38.7 (33.0-46.5)	56.0 (44.0-73.7)^b^	73.5 (55.0-87.0)^b,c^

IFN-γ	4.0 (3.0-4.0)	3.0 (2.0-3.0)^b^	5.0 (4.0-6.0)^b,d^

TNF-α	2.0 (2.0-3.0)	2.0 (1.0-2.0)^a^	2.0 (2.0-3.0)^d^

TGF-β1	342 (285-388)	364 (307-413)	383 (331-418)

IP-10	198 (142-327)	327 (222-442)^b^	688 (434-928)^b,d^

MCP-1	153 (87.5-233)	79.5 (52.5-110)^b^	189 (120-283)^d^

ICAM-1	6638 (6143-7205)	6789 (6201-7672)	8132 (7413-8808)^b,d^

VCAM-1	6151 (5767-6564)	6157 (5633-6617)	7386 (6913-7709)^b,d^

Data are presented as median (25-75 percentile)

IL: interleukin; IL-1ra: IL-1 receptor antagonist; IFN: interferon; TNF: tumor necrosis factor; TGF: transforming growth factor; IP: interferon-γ-inducible protein; MCP: monocyte chemotactic protein; ICAM: intercellular adhesion molecule; VCAM: vascular cell adhesion molecule

^a ^p < 0.05 versus healthy non-pregnant women

^b ^p < 0.001 versus healthy non-pregnant women

^c ^p < 0.05 preeclamptic patients versus healthy pregnant women

^d ^p < 0.001 preeclamptic patients versus healthy pregnant women

**Figure 1:**
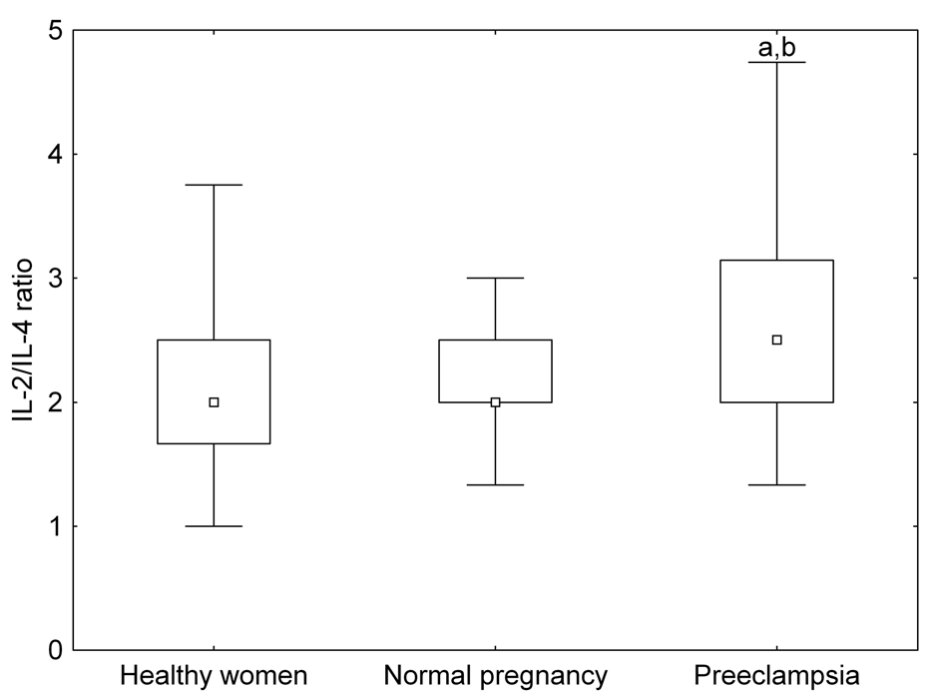
**IL(interleukin)-2/IL-4 ratios of healthy non-pregnant and pregnant women and preeclamptic patients**. Middle point: median; Box: interquartile range (25-75 percentile); Whisker: range (excluding outliers); ^a ^p < 0.001 versus healthy non-pregnant women; ^b ^p < 0.05 versus healthy pregnant women

**Figure 2:**
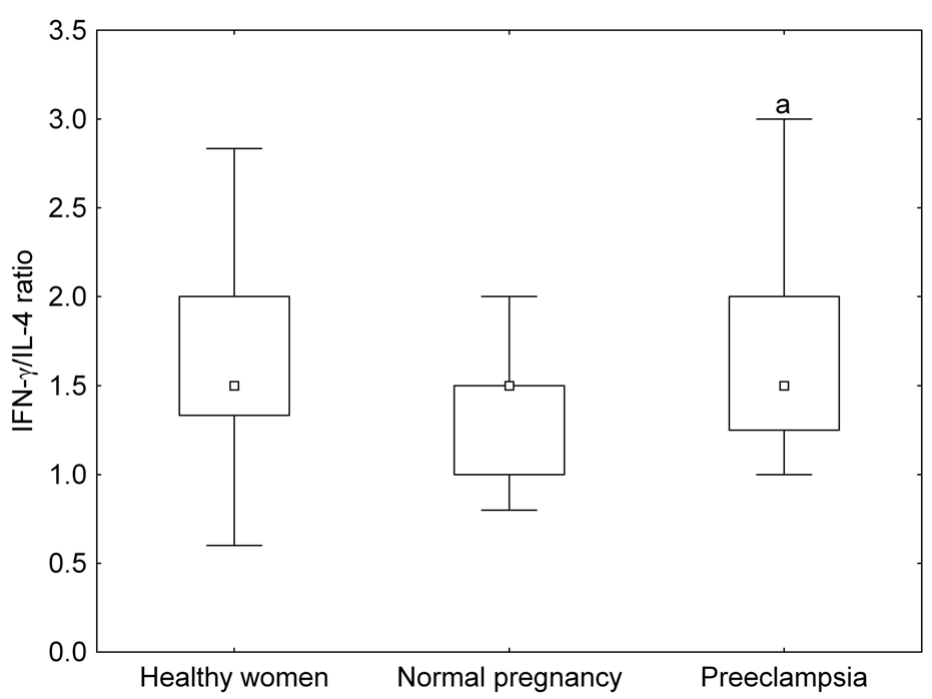
**IFN(interferon)-γ/IL(interleukin)-4 ratios of healthy non-pregnant and pregnant women and preeclamptic patients**. Middle point: median; Box: interquartile range (25-75 percentile); Whisker: range (excluding outliers); ^a ^p < 0.05 versus healthy pregnant women

**Figure 3:**
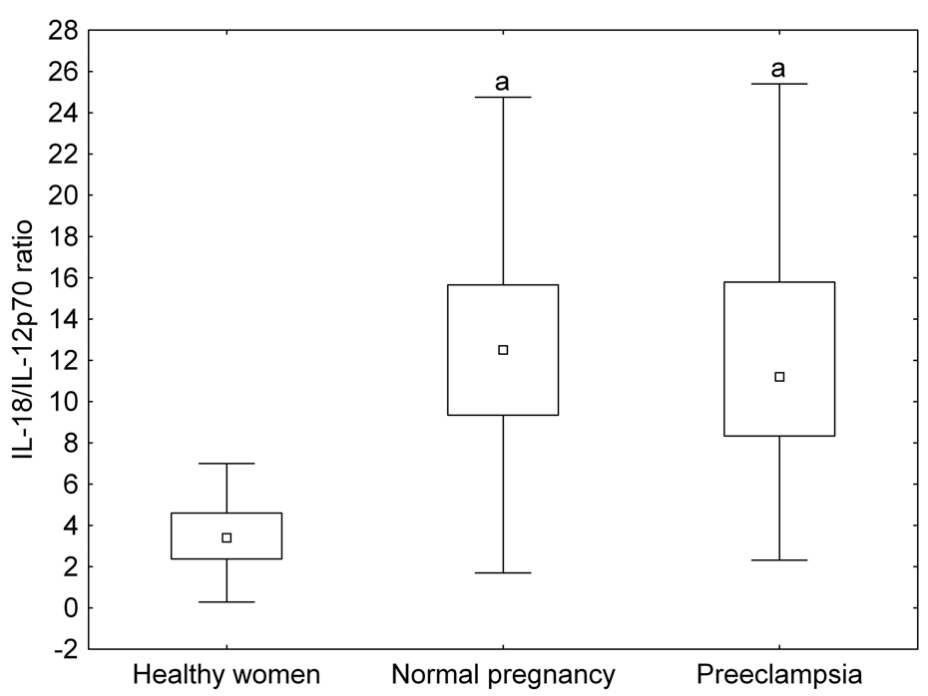
**IL(interleukin)-18/IL-12p70 ratios of healthy non-pregnant and pregnant women and preeclamptic patients**. Middle point: median; Box: interquartile range (25-75 percentile); Whisker: range (excluding outliers); ^a ^p < 0.001 versus healthy non-pregnant women

**Figure 4:**
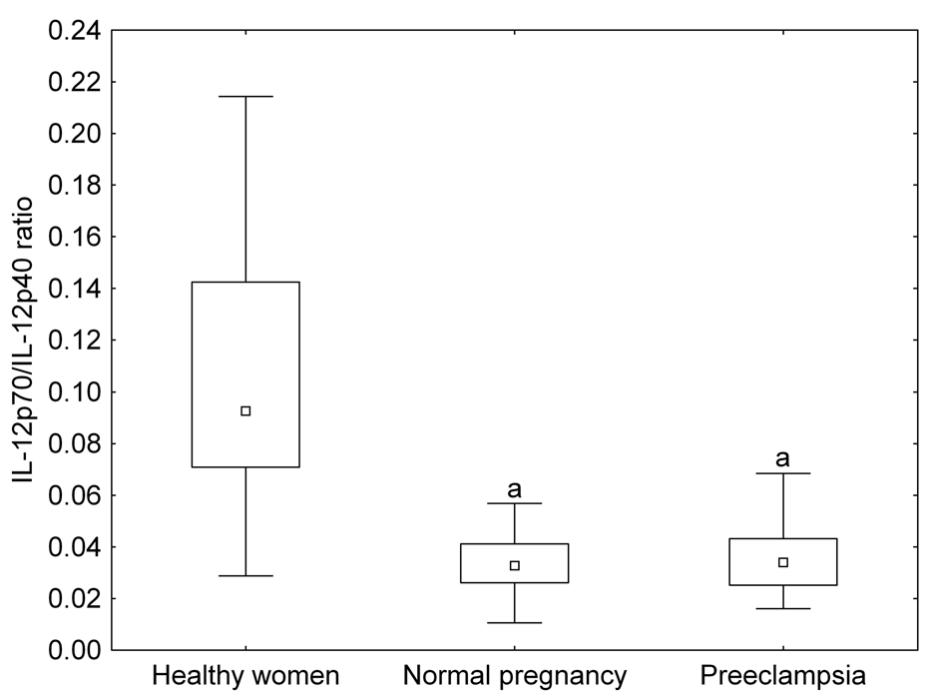
**IL(interleukin)-12p70/IL-12p40 ratios of healthy non-pregnant and pregnant women and preeclamptic patients**. Middle point: median; Box: interquartile range (25-75 percentile); Whisker: range (excluding outliers); ^a ^p < 0.001 versus healthy non-pregnant women

There were no significant differences in the ratios of IL-2 to IL-4 and IFN-γ to IL-4 between healthy non-pregnant and pregnant women, whereas these ratios were significantly increased in preeclamptic patients as compared to healthy pregnant women (Figure 1, 2). On the contrary, IL-18/IL-12p70 ratios were significantly higher, while IL-12p70/IL-12p40 ratios were significantly lower in healthy pregnant than in non-pregnant women, but they showed the same level in preeclamptic patients compared with healthy pregnant women (Figure 3, 4).

In the group of preeclamptic patients, no statistically significant differences were found in serum levels of the measured cytokines, chemokines and adhesion molecules between patients with mild and severe preeclampsia, between patients with late and early onset of the disease, or between preeclamptic patients with and without fetal growth restriction (data not shown).

### Relationship of serum cytokine, chemokine and adhesion molecule levels of the study subjects with their clinical characteristics and laboratory parameters

We also investigated whether serum cytokine, chemokine and adhesion molecule levels of the study participants were related to their clinical features and laboratory parameters by calculating the Spearman rank order correlation coefficients (continuous variables) or by the Mann-Whitney *U*-test (categorical variables). In healthy non-pregnant women, serum IL-6 and TNF-α concentrations correlated significantly with CRP levels (Spearman R = 0.28 and 0.29, respectively, p < 0.05). In the group of healthy pregnant women, we found statistically significant negative correlations between serum IL-2 and IFN-γ concentrations and gestational age at delivery (R = -0.27 and -0.29, respectively, p < 0.05). A significant positive correlation was observed between IL-6 and CRP levels of healthy pregnant women (R = 0.45, p < 0.05), while their TGF-β1 and malondialdehyde concentrations correlated inversely with each other (R = -0.38, p < 0.05). Serum IP-10 levels of healthy pregnant women showed significant positive correlations with serum creatinine levels (R = 0.53, p < 0.05), as well as with plasma levels of VWF:Ag (R = 0.54, p < 0.001) and fibronectin (R = 0.42, p < 0.05), while a significant inverse correlation with fetal birth weight (R = -0.38, p < 0.05). Furthermore, there were significant positive correlations between their serum MCP-1 concentrations and serum creatinine (R = 0.39, p < 0.05), as well as plasma fibronectin levels (R = 0.48, p < 0.001). Significant correlations between inflammatory variables of preeclamptic patients and their clinical characteristics and laboratory parameters are presented in Table 4. There was no other relationship between serum cytokine, chemokine and adhesion molecule levels of the study subjects and their clinical features and measured laboratory parameters in either study group.

**Table 4:** Significant correlations of serum cytokine, chemokine and adhesion molecule levels of preeclamptic patients with their clinical characteristics and laboratory parameters

	Correlation coefficient	Statistical significance (p value)
IP-10 & BUN level	0.26	<0.05

IP-10 & creatinine level	0.43	<0.05

IP-10 & AST activity	0.46	<0.001

IP-10 & ALT activity	0.38	<0.05

IP-10 & LDH activity	0.38	<0.05

IP-10 & VWF:Ag level	0.35	<0.05

IP-10 & fibronectin level	0.37	<0.05

MCP-1 & systolic blood pressure	0.27	<0.05

MCP-1 & CRP level	0.27	<0.05

MCP-1 & malondialdehyde level	0.27	<0.05

ICAM-1 & bilirubin level	0.32	<0.05

ICAM-1 & AST activity	0.32	<0.05

ICAM-1 & LDH activity	0.37	<0.05

ICAM-1 & CRP level	0.30	<0.05

ICAM-1 & malondialdehyde level	0.31	<0.05

VCAM-1 & BUN level	0.30	<0.05

VCAM-1 & creatinine level	0.44	<0.001

VCAM-1 & AST activity	0.40	<0.05

VCAM-1 & LDH activity	0.56	<0.001

VCAM-1 & fibronectin level	0.41	<0.05

IP: interferon-γ-inducible protein; BUN: blood urea nitrogen; AST: aspartate aminotransferase; ALT: alanine aminotransferase; LDH: lactate dehydrogenase; VWF:Ag: von Willebrand factor antigen; MCP: monocyte chemotactic protein; CRP: C-reactive protein; ICAM: intercellular adhesion molecule; VCAM: vascular cell adhesion molecule

## Discussion

In this study, we determined circulating levels of several cytokines, chemokines and adhesion molecules in healthy non-pregnant and pregnant women and preeclamptic patients by high-throughput multiplex suspension array technology. Except for serum IL-1β and TGF-β1 levels, all of the measured inflammatory variables differed significantly among the three study groups. Simultaneous measurement of several markers of disease processes enabled us to explore their role in the pathogenesis of preeclampsia.

Normal pregnancy is characterized by a shift towards Th2-type immunity and the inhibition of cytotoxic Th1 immune responses, which could be harmful to the fetus (reflected by the inverse correlation of serum IL-2 and IFN-γ levels with gestational age at delivery in our healthy pregnant women) [20]. IL-18 and IL-12 are the key cytokines regulating Th1/Th2 balance. IL-18 alone can induce Th2-type immunity, but in the presence of IL-12, IL-18 stimulates Th1-mediated immune responses [21]. Indeed, the ratios of IL-18 to IL-12 secreted by PBMCs have been reported to be significantly increased in normal pregnancy [22]. In healthy pregnant women, the relative abundance of circulating IL-18 over IL-12 expressed by the increased serum IL-18/IL-12p70 ratios observed in our study, as well as the relative deficiency of the bioactive IL-12p70 in relation to IL-12p40 (its competitive inhibitor) reflected by the decreased serum IL-12p70/IL-12p40 ratios, might favour Th2-type immunity. In our preeclamptic patients, serum IL-12p70 levels were significantly higher as compared to healthy pregnant women. Although circulating IL-18 and IL-12p40 levels were also elevated yielding similar IL-18/IL-12p70 and IL-12p70/IL-12p40 ratios as in normal pregnancy, the relative abundance of circulating IL-2 and IFN-γ over IL-4 - as shown by the increased serum IL-2/IL-4 and IFN-γ/IL-4 ratios - might provide a Th1-biased systemic environment in preeclampsia.

In addition to changes in Th1/Th2 balance, several other soluble inflammatory variables were also altered in normal pregnancy and preeclampsia. Circulating levels of the pro-inflammatory cytokines IL-6 and TNF-α, the chemokines IL-8, IP-10 and MCP-1, as well as the adhesion molecules ICAM-1 and VCAM-1, were raised in preeclampsia compared with healthy pregnancy, resulting in an overall pro-inflammatory systemic environment. Elevated circulating IL-1 receptor antagonist concentrations in preeclampsia reflect increased activity of the pro-inflammatory cytokines IL-1α and β, which have a very short half-life in the circulation, and therefore it is difficult to detect a difference in their serum levels [23]. The increase in levels of the immunoregulatory cytokine IL-10 in our preeclamptic patients is in line with previous findings and might be a compensatory phenomenon [24]. On the other hand, the changes in circulating cytokine profile in our healthy pregnant group were - at least in part - anti-inflammatory as shown by the decreased IL-1ra, TNF-α and MCP-1 concentrations relative to non-pregnant women. However, decreased serum IL-10 and increased IP-10 levels found in our healthy pregnant women might drive pro-inflammatory responses. Indeed, the third trimester of normal pregnancy seems to be a controlled state of systemic inflammation, as expressed also by the elevated serum CRP levels in our study [25]. Interestingly, a state of controlled inflammation at the feto-maternal interface in early pregnancy with production of pro-inflammatory cytokines and chemokines is thought to be beneficial for trophoblast invasion [26,27]. Although serum concentrations of TGF-β1 did not differ among our study groups, elevated levels of its soluble co-receptor, endoglin, have been observed in preeclampsia previously [28]. Soluble endoglin impairs binding of TGF-β1 to its receptors and downstream signalling, leading to dysregulated TGF-β signalling in the vasculature.

The maternal systemic inflammatory response characteristic of both the third trimester of normal pregnancy and - in an excessive form - preeclampsia involves an acute-phase reaction as well as systemic oxidative stress, and circulating cytokines are central to these processes [29]. Pro-inflammatory cytokines, primarily IL-6, can induce an acute-phase response [30]. Furthermore, cytokines can cause the release of oxygen free radicals, whereas reactive oxygen metabolites can up-regulate the genes that code for pro-inflammatory cytokines and adhesion molecules [31]. Indeed, serum IL-6 (and TNF-α) concentrations correlated with CRP levels in our healthy non-pregnant and pregnant groups. The inverse correlation between TGF-β1 and malondialdehyde levels of our healthy pregnant women indicates that TGF-β1 could inhibit lipid peroxide production in normal pregnancy. Interestingly, serum MCP-1 and ICAM-1 concentrations showed significant positive correlations with CRP and malondialdehyde levels in the group of preeclamptic patients, which implies that recruitment and adhesion of leukocytes to endothelial cells are central features of the generalized intravascular inflammatory reaction and oxidative stress observed in preeclampsia. The correlation of MCP-1 and ICAM-1 concentrations with blood pressure values and liver function parameters, respectively, suggests that these cytokines and the inflammatory processes they mediate might contribute to the development of hypertension and hepatocellular injury in this pregnancy-specific disorder.

Cytokines, chemokines and adhesion molecules could be potential mediators of endothelial dysfunction, which is a hallmark of the maternal syndrome of preeclampsia. Therefore, we examined whether these inflammatory variables were related to the markers of endothelial activation (von Willebrand factor antigen) and injury (fibronectin). In this study, significant correlations were found between IP-10, MCP-1 and VCAM-1 levels and endothelial markers in normal pregnancy and preeclampsia. Certain organs with fenestrated (discontinuous) endothelium, such as the kidney (glomeruli), liver (sinusoids) and brain (choroid plexus) are disproportionally affected in preeclampsia. Interestingly, serum IP-10, MCP-1 and VCAM-1 concentrations were also related to renal and liver function parameters in our study. These findings denote the central role of these inflammatory molecules in mediating endothelial damage. IP-10 showed the strongest association with endothelial dysfunction in our healthy pregnant women and preeclamptic patients. Indeed, IP-10 (CXCL10) has pro-inflammatory and anti-angiogenic properties, and this chemokine has been proposed to be a potential link between inflammation and anti-angiogenesis in preeclampsia [32]. The inverse correlation of IP-10 levels with fetal birth weight of healthy pregnant women suggests its inhibitory role in placental angiogenesis. Although TNF-α can also elicit endothelial cell dysfunction and injury, no significant relationship was observed between its serum concentration and endothelial markers in this study [33]. Nevertheless, we did not measure levels of soluble TNF receptors, which have a longer half-life than TNF-α and, thus, are thought to be more reliable markers of TNF-α activity.

The placenta is supposed to be a potential source of circulating inflammatory cytokines in preeclampsia [34]. Interestingly, syncytiotrophoblast sheds placental debris into the maternal circulation in preeclampsia with elevated amounts. The mass of this trophoblast debris can be assessed by the measurement of copies of cell-free fetal DNA in the maternal plasma [35,36]. However, circulating levels of cytokines and other inflammatory molecules did not show a significant correlation with those of cell-free fetal DNA in our study, indicating that trophoblast deportation process may not substantially contribute to the elevated circulating concentrations of inflammatory molecules. Others have also questioned that the placenta is the major source of pro-inflammatory cytokines in the circulation of preeclamptic women [37]. Indeed, dysfunctional maternal endothelial cells and activated circulating leukocytes could also release inflammatory molecules into the blood in this disorder. Additionally, there is a strong genetic influence on cytokine production. Therefore, genetic factors might also account - at least partly - for the abnormal cytokine profile observed in preeclampsia [4,38-41].

In this study, the similar cytokine profile of preeclamptic patients regardless of the severity, the time of onset of the disease or the presence of fetal growth restriction might be explained by the multifactorial etiology of preeclampsia. Several genetic, behavioural and environmental factors need to interact to produce the complete picture of this pregnancy-specific disorder. Our research group reported various genetic and soluble factors that were associated with the severity or complications of preeclampsia, including HELLP syndrome and fetal growth restriction [42-45]. Nevertheless, it is also possible that the relatively small sample size of this study prevented to detect an effect in the subgroup analyses.

## Conclusions

According to our findings, preeclampsia was associated with an overall pro-inflammatory systemic environment. Elevated amounts of pro-inflammatory cytokines, chemokines and adhesion molecules in the maternal circulation might play a central role in the excessive systemic inflammatory response, as well as in the generalized endothelial dysfunction characteristics of the maternal syndrome of preeclampsia.

## Authors' contributions

ASZ collected data and drafted the manuscript. JR participated in the design of the study. LL determined cell-free fetal DNA. GB carried out multiplex suspension array measurements. AM conceived of the study, participated in its design and coordination, performed statistical analyses and helped to draft the manuscript. All authors read and approved the final manuscript.
